# Examiners of the London University

**Published:** 1839-07

**Authors:** 


### EXAMINERS OF THE LONDON UNIVERSITY.

IN a preceding article, on Medical Education and Reform, we have given strong proofs of our being sincere and zealous friends of the University of London; and we have now much pleasure in adding that we think the medical members of the senate are entitled to the gratitude of the profession for the admirable curriculum of education, preliminary and professional, which they have established for the candidates for university degrees. We need hardly say, then, that it is with extreme regret that we have to express our conviction that the University, in one of its first steps towards carrying its regulations into effect, has committed a serious mistake. As the terms of the curriculum leave it, in a very considerable degree, optional with the candidates to obtain the requisite knowledge where and in what manner they please, it follows, as a matter of course, that, in order to stamp the degrees with anything like distinction, or even with a current value, the EXAMINATIONS should possess a high character for extensiveness, strictness, and impartiality, and the EXAMINERS should be men of acknowledged eminence. Now, we presume to doubt if the senate have kept sufficiently in view these important truths, in the recent election of examiners, more particularly in the departments of Medicine and Surgery. While freely admitting the unexceptional personal and professional character of the gentlemen chosen in these departments, we refuse to acknowledge them as possessing that degree of eminence in either literature or science which can give any particular distinction to the tribunal in which they are judges. We are glad, for the sake of the University, that these appointments are only for a single year; and we warn the Senate that, if the golden rule of *Detur Optimo* be not rigidly adhered to in all their future elections, the glory of the Institution will be eclipsed in its very dawn.

